# The effects of extremes of pH on the growth and transcriptomic profiles of three haloarchaea

**DOI:** 10.12688/f1000research.4789.2

**Published:** 2014-08-29

**Authors:** Aida Moran-Reyna, James A. Coker

**Affiliations:** 1 Department of Biology, University of Alabama at Birmingham, Birmingham, AL, 35294-1170, USA; 2 The Graduate School, University of Maryland, University College, Largo, MD, 20774, USA

## Abstract

The halophilic archaea (haloarchaea) live in saline environments, which are found across the globe.  In addition to salinity, these niches can be quite dynamic and experience extreme conditions such as low oxygen content, radiation (gamma and UV), pH and temperature.  However, of all the naturally occurring stresses faced by the haloarchaea, only one, pH, has not been previously investigated in regard to the changes induced in the transcriptome. Therefore, we endeavored to determine the responses in three haloarchaea: *Halorubrum lacusprofundi* (Hla), *Haloferax volcanii* (Hvo), and *Halobacterium* sp. NRC-1 (NRC-1) to growth under acidic and alkaline pH. Our observations showed that the transcriptomes of Hvo and NRC-1 regulated stress, motility, and ABC transporters in a similar manner, which is in line with previous reports from other prokaryotes when grown in an acidic environment.  However, the pattern for Hla was more species specific. For alkaline stress, all three haloarchaea responded in a manner similar to well-studied archaea and bacteria showing the haloarchaeal response was general to prokaryotes. Additionally, we performed an analysis on the changes in the transcriptomes of the three haloarchaea when shifting from one pH extreme to the other. The results showed that the transcriptomes of all three haloarchaea respond more similarly when moving from alkaline to acidic conditions compared to a shift in the opposite direction. Interestingly, our studies also showed that individual genes of multiple paralogous gene families ( *tbp*, *tfb*, *orc*/ *cdc6*, etc.) found in the haloarchaea were regulated under specific stresses thereby providing evidence that they modulate the response to various environmental stresses. The studies described here are the first to catalog the changes in the haloarchaeal transcriptomes under growth in extreme pH and help us understand how life is able to thrive under all conditions present on Earth and, if present, on extraterrestrial bodies as well.

## Introduction

The halophilic archaea (haloarchaea) live in saline environments, such as the Great Salt Lake in Utah, the Dead Sea, and solar salterns. They belong to a single order (Halobacteriales), which consists of a single family (Halobacteriaceae), making them genetically similar to each other ^1^. Many haloarchaea are facultative aerobes as extreme saline environments often have low oxygen solubility ^2^. Haloarchaea have adapted to survive in their saline environments by employing a tactic called “salting in” whereby they selectively uptake K ^+^ and Cl ^-^ ions to a concentration of greater than three molar. To keep their proteins from precipitating in these high ionic strength conditions, haloarchaea have also adapted a primarily acidic proteome with proteins containing most of their negative charges on their surface ^3^. The negative charges on the surface help orient water molecules to keep the proteins hydrated thereby stopping the possibility of precipitation.

Although saline environments are often studied in relation to salt content, they have been shown to have other extreme conditions: temperature, gamma radiation, oxygen sensitivity, UV, and pH, which all affect the adaptation and survival of the species in these niches. Of these conditions, transcriptomic responses to temperature ^4^, salinity ^4^, oxygen requirements ^5^, and radiation ^6, 7^ are well studied. For extremes of temperature, it has been previously shown that haloarchaea over-express chaperones ( *hsp* and *csp*), DNA binding proteins ( *hpyA*), and other previously characterized temperature stress genes/proteins ^8^. For high energy (gamma) radiation, the haloarchaea have been shown to be the second most naturally resistant life form on the planet, slightly less resistant than *Deinococcus radiodurans* ^7^. They are also able to withstand UV intensities about 100× higher than those experienced by organisms on the surface of Earth (>250 J/m ^2^ vs. 2–3 J/m ^2^) ^6, 9, 10^. Haloarchaea are able to survive these conditions through an overexpression of DNA binding/repair proteins ^7, 9– 11^.

Although previous transcriptomic studies of the haloarchaea have not focused on pH, the response from other prokaryotic organisms such as *Escherichia coli* and *Bacillus subtilis* has been thoroughly examined ^12– 14^. These studies are important because of the role that pH plays in disease and its influence on the passage of prokaryotic species through and/or colonization of specific areas of the human body (e.g. acidic conditions in the stomach and beginning of the small intestine and alkaline conditions in the intestines). *E. coli*, when the environment is acidic, tends to use pathways that consume acids as well as increase the production of the inner membrane protein YagU and hydrogenases; however, when the environment is alkaline metabolic genes are up-regulated as are pathways that result in the production of acids to neutralize the effect of alkaline pH in the external environment ^12, 14^. For *B. subtilis*, acidic stress is of extreme importance because it leads to up-regulation of spore germinating genes, which allow the organism to survive in the hostile conditions of the stomach ^13, 15^.

The best studied extremophilic organisms in regard to the effects of pH are the acidophiles and the alkaliphiles. These studies have primarily shown that these organisms survive *via* passive and active responses. Passive responses involve the modification of the cell wall/membrane to keep out excess H ^+^ and OH ^-^ ions ^16, 17^. Active responses primarily involve pumping in/out specific ions. For alkaliphiles this is accomplished with the use of Na ^+^/H ^+^ antiporters where Na ^+^ is pumped out of the cell and H ^+^ is pumped in, as well as an up-regulation of pathways that produce acid ^16^; however, the active processes used by acidophiles are not well known.

Insights into the mechanisms of archaeal adaptations to the extremes of high salinity ^2^ and acidity ^18^ or alkalinity ^19^ have been gained via genome sequencing projects by addressing each issue separately. However, more in depth knowledge of dual extremes is lacking. Haloarchaea are one of the few groups of Archaea that have been isolated from both extremes of the pH scale ^19, 20^ and therefore represent a novel group of organisms that are ideal subjects for studies to determine the transcriptomic responses from multiple extremes. On the lower end of the pH scale, haloarchaea are commonly found in acidic lakes such as Lake Afrera, Ethiopia, and Lake Aerodrome and Lake Brown in Western Australia ^20^. On the higher end of the pH scale, the haloarchaea are commonly found in alkaline lakes such as Lake Natron and Lake Magadi in the Great Rift Valley in Africa as well as Mono Lake in California ^19, 21^.

Therefore, we undertook the following study to determine the changes in transcriptomes of three well-studied haloarchaea: *Halorubrum lacusprofundi, Haloferax volcanii*, and *Halobacterium* sp. NRC-1, to understand the effects on the transcriptome associated with growth of haloarchaea at extremes of pH. These organisms were selected primarily because they have a fully sequenced genome and they can be easily grown and manipulated within the laboratory. However, they also represent a range of temperatures, 30°C (Hla) to 49°C (Hvo), and salinity, 2.5 (Hvo) to 4.2 M (NRC-1), optima. They are also naturally found across the glove and as we show below are able to grow in both acidic and alkaline conditions. As a result, the data gained from these studies will help determine if the transcriptomic responses are merely species specific, representative of the haloarchaea in general, or a part of a phylogenetically wider response to pH stress.

## Materials and methods

### Culturing

*Halorubrum lacusprofundi* DSMZ 5036 (Hla) cultures were grown in standard Artificial Deep Lake medium at 30°C ^22^. *Haloferax volcanii* DS2 (Hvo) cultures were grown in standard HV-YPC medium at 49°C ^23^. *Halobacterium* sp. NRC-1 ATCC 700922 (NRC-1) cultures were grown in standard CM ^+^ medium at 42°C ^2^. Each organism was grown in an Innova 42R platform shaker at 220 rpm. For growth in varying pH, the above media were prepared as described with the pH altered to 4.4, 5.4, 6.4, 7.4 and 8.4 for Hla, 4.5, 5.5, 6.0, 6.5, 7.5, 8.0 and 8.5 for Hvo, and 4.2, 5.2, 6.2, 7.2, 8.2 and 9.2 for NRC-1 using HCl or NaOH. Growth curves were measured in 50 mL cultures by removing 1 mL aliquots at various time points and measuring the optical density (OD _600_) in a Shimadzu UV-160 spectrophotometer. The pH of the growing cultures was checked with pH Test Strips (Ricca Chemical) each time an aliquot was removed for an optical density measurement. Each growth curve was replicated four times for each condition. The doubling times of each organism, under each pH condition, were derived by calculating the line of least squares during the exponential phase of growth and the significance checked by ANOVA.

### DNA microarray design

Oligomer (60-mer) probes used in our arrays were designed using the program PICKY ( http://www.complex.iastate.edu) with the following criteria: selection_left_begin = 0, selection_right_end = 0, maximum_oligo_size = 70, minimum_oligo_size = 60, maximum_match_len = 15, minimum_match_len = 13, maximum_gc_content = 70, minimum_gc_content = 50, candidates_per_gene = 5, probes_per_gene = 4, minimum_similarity = 75, minimum_temp_separation = 10. For each organism, the arrays contained 13 probes for each of the 3538 annotated genes in Hla, eight probes for each of the 4020 genes in Hvo, and 17 probes for each of the 2524 genes in NRC-1. Oligonucleotide arrays were *in situ* synthesized by Agilent using ink-jet technology and used for transcriptome analysis of all three organisms. Signal intensities with a dynamic range in excess of three orders of magnitude were found allowing simultaneous analysis of low and high-intensity features.

### Microarray sample preparation and scanning

For the microarray experiments for each organism, six cultures were grown: two under acidic pH, two under alkaline pH, and two under optimal pH. These cultures were grown in the same manner as those used for the growth in varying pH described above. All cultures for three organisms studied were harvested at late exponential phase (OD _600_ = 0.9–1.0) for microarray analysis. Total nucleic acids (DNA and RNA) were purified from the cultures using the Agilent total RNA isolation mini kit. Total nucleic acids were then incubated with RNase-free DNase I (New England Biolabs) to digest the genomic DNA. RNA was then purified using the Agilent total RNA isolation mini kit. RNA was pooled from both cultures for cDNA synthesis. cDNA was prepared for control (optimal pH) and experimental (acidic/alkaline pH) samples using fluorescently labeled *Cy*3-dCTP and *Cy*5-dCTP, respectively. Labeled cDNAs were sent to the Interdisciplinary Center for Biotechnology Research (ICBR) at the University of Florida for hybridization and scanning. Concentrations of cDNA were measured using a Nanodrop (ND-1000) spectrophotometer (NanoDrop Technologies, Wilmington, DE) at ICBR. Hybridization was performed as recommended by Agilent and as previously described ^8, 24^ using 1 µg (0.5 µg for experimental and control) of total cDNA per microarray. Microarray analysis was performed in duplicate for technical replicates and slides were scanned for *Cy*-3 and *Cy*-5 signals with an Agilent DNA-microarray scanner as previously done ^5, 25^.

### Microarray data processing

Probe signals were extracted and initial analysis was done with the Agilent Feature Extraction Software, where signal from each channel was normalized using the LOWESS algorithm to remove intensity-dependent effects within the calculated values. The data were further parsed using Agilent’s Genespring GX software. Genes showing greater than 2-fold change in transcript abundance in at least two of the replicates with an illuminant intensity greater than 7 and less than 14 were selected for further analysis, as described previously ^8, 24, 26^.

## Results and discussion

We employed three well-studied haloarchaea: *Halorubrum lacusprofundi* (Hla), *Haloferax volcanii* (Hvo), and *Halobacterium* sp. NRC-1 (NRC-1), to understand the effects that pH stress plays on the transcriptome of haloarchaea. These organisms were selected due to the depth of knowledge in relation to adaption/survival to other naturally occurring stresses ^8, 27– 30^. This element was key to tease out differences between the general stress response and pH specific responses. We also chose three organisms, representing a range of temperature and salinity optima, to ascertain if the response to pH was specific to an organism or the Halobacteriaceae in general. Further, we compared our data to previous studies to determine if the response observed was part of a wider Prokaryotic response or common to all three Domains of Life.

### Growth under extremes of pH

The growth optimum for the Halobacteriaceae is around pH 7 ^2, 22, 23^. Specifically, for the organisms used in this study, it is pH 7.4, 7.5, and 7.2 for *Halorubrum lacusprofundi*, *Haloferax volcanii*, and *Halobacterium* sp. NRC-1, respectively. In order to determine the changes in gene transcripts during growth at pH extremes we first determined the range of growth for each organism as it was previously unknown.

**Figure 1.**
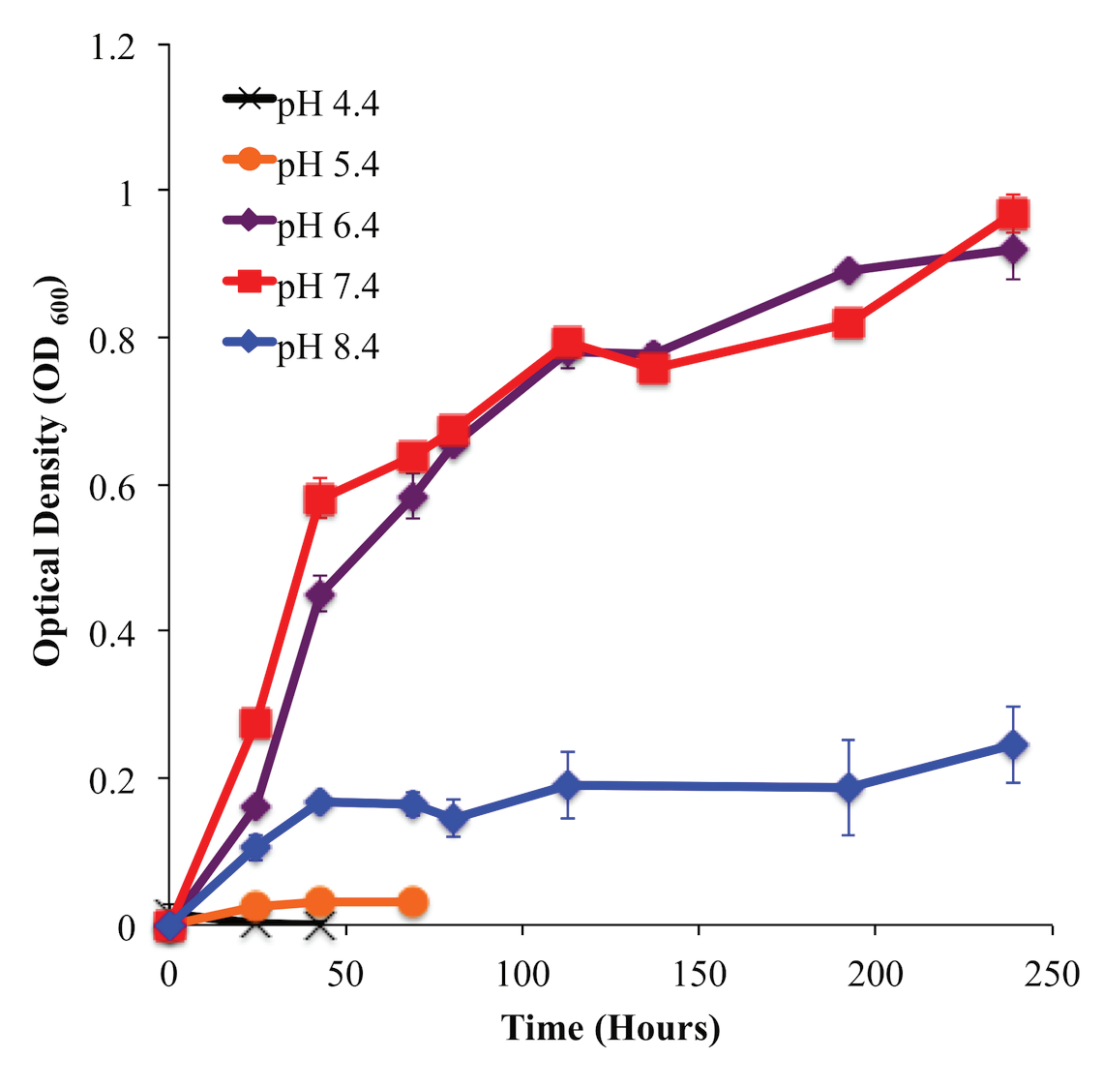
Growth Curve of Hla cultures grown under acidic and alkaline conditions. Growth of *Halorubrum lacusprofundi* at pH 4.4 (black ×s), pH 5.4 (light green circles), pH 6.4 (light blue diamonds), pH 7.4 (red squares), and pH 8.4 (blue diamonds). Values for the symbols are the average of all replicates and the error bars represent the standard deviation at each time point.

*Halorubrum lacusprofundi –* Growth was observed from pH 6.4 to 8.4 ( Figure 1). The doubling time at pH 6.4 was around 64 hours and at 8.4 was 77 hours, both well above the 44 hours at pH 7.4 (optimum). Growth was tested but not observed at pH 4.4 or 5.4.

**Figure 2.**
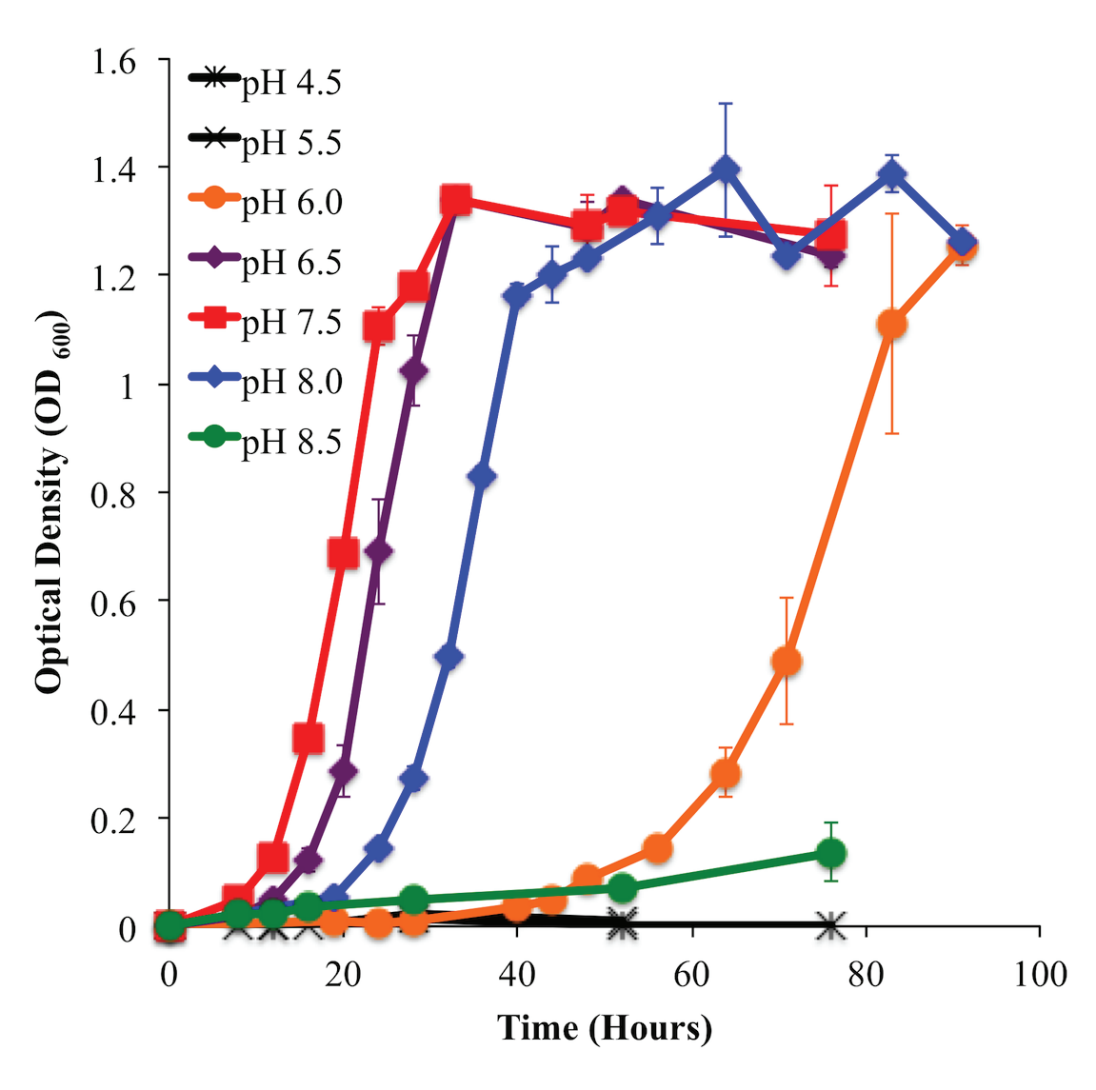
Growth Curve of Hvo cultures grown under acidic and alkaline conditions. Growth of *Haloferax volcanii* at pH 4.5 (black asterisks), pH 5.5 (black ×s), pH 6.0 (light green circles), pH 6.5 (light blue diamonds), pH 7.5 (red squares), pH 8.0 (blue diamonds), and pH 8.5 (green circles). Values for the symbols are the average of all replicates and the error bars represent the standard deviation at each time point.

*Haloferax volcanii –* Growth was observed from pH 6.0 to 8.0 ( Figure 2). Doubling time at pH 6.0 was about 7.0 hours while at pH 6.5 it almost halved to about 3.7 hours. At the typical laboratory growth pH of 7.5 the doubling time was about 3.2 hours. Under alkaline growth conditions (pH 8.0), the doubling time was about 4.0 hours. Growth was tested at pH 4.5, 5.5 and 8.5 but not observed.

**Figure 3.**
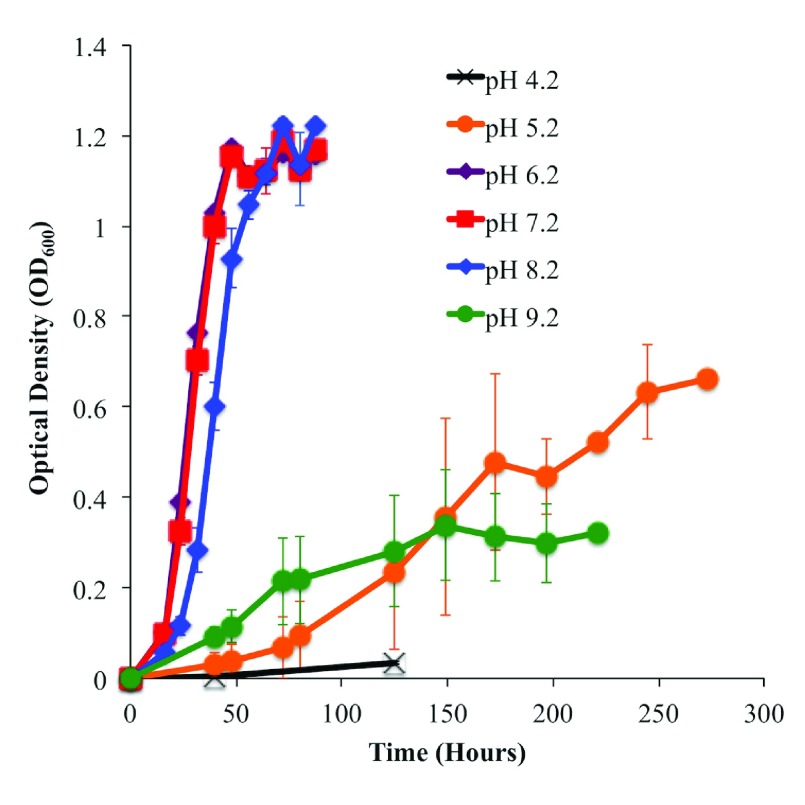
Growth Curve of NRC-1 cultures grown under acidic and alkaline conditions. Growth of *Halobacterium* sp. NRC-1 at pH 4.2 (black ×s), pH 5.2 (light green circles), pH 6.2 (light blue diamonds), pH 7.2 (red squares), pH 8.2 (blue diamonds), and pH 9.2 (green circles). Values for the symbols are the average of all replicates and the error bars represent the standard deviation at each time point.

*Halobacterium* sp. NRC-1 – Growth was observed from pH 5.2 to 9.2 ( Figure 3). Doubling times at pH 8.2, 7.2 and 6.2 were about 8.6, 8.6, and 9.6 hours, respectively. Growth at pH 5.2 was considerably slower and had a doubling time around 75 hours. Growth at pH 9.2 was the slowest recorded with a doubling time of about 150 hours. Growth was tested but not observed at pH 4.2.

The pH range for *Halorubrum lacusprofundi* and *Haloferax volcanii* were well within the ranges previously reported for other species from the same genera ^31^. However, the range for *Halorubrum* sp. NRC-1 was a little bigger (0.5 pH units) than that reported for other species within the genus ^31^. NRC-1 also had the widest range (4 pH units) of growth of the three haloarchaea studied. Doubling times for the organisms under their optimal conditions were similar to those previously published ^2, 22, 30^. Additionally, an analysis of variance (ANOVA) test was performed for the growth curves for each organism and in each case the data was found to be significant (data not shown).

### Microarray analysis and responses of individual organisms

Once the pH range of growth was determined, we grew each of the three organisms at its optimum pH and at the most acidic and alkaline pH where growth was observed. This was done in an effort to maximize the potential differences (if any) in the transcriptomes. We designed all three microarrays (one per organism) used for these experiments and each incorporated at least eight probes per gene in the entire genome. Hybridizations were designed so a positive fold-change value would correspond with an up-regulation of a gene transcript in the experimental condition (growth in acidic or alkaline conditions) and a negative value would correspond with a down-regulation of a gene transcript.

### Alterations in the transcriptomes under growth in alkaline conditions

*Halorubrum lacusprofundi* – For *H. lacusprofundi*, microarray analysis showed that transcripts from 532 genes were significantly up-regulated and 608 were down-regulated at pH 8.4, compared to pH 7.4 ( Table 1). For the up-regulated gene transcripts, the largest percent were members of COG group E (amino acid transport and metabolism group) and the lowest were COG group D (cell cycle control, cell division, chromosome partitioning). For the down-regulated gene transcripts, the largest percent were members of COG group R (general function prediction only) and the lowest was COG group M (cell wall/membrane/envelope biogenesis). About 65% of the up-regulated and 45% of the down-regulated transcripts in Hla were from genes without an assigned function (no COG, general function, and unknown function), which is typical for the Archaea.

**Table 1.** Transcripts regulated under growth in alkaline conditions.

Organism(s)	Number of genes shared Up- regulated	Number of genes shared Down- regulated
Hla	532	608
Hvo	326	1581
NRC-1	471	72
Hla and NRC-1	89	15
Hla and Hvo	60	311
NRC-1 and Hvo	62	41
Hla, Hvo, NRC-1	36	15

Despite the large number of unknowns, there were several transcripts/genes of interest in Hla. Of these, most of the up-regulated transcripts fell into the following groups: tRNA, metabolism, and stress genes. For the tRNA transcripts, all 27 showed an up-regulation from 2- to 11-fold. For metabolism transcripts, these included phosphoenolpyruvate (PEP) carboxylase and cytochrome P450, with its corresponding ferridoxin. For the stress genes, transcripts of genes such as the small heat shock ( *hsp20* family), Cpn60/TCP-1, and universal stress ( *uspA*) were regulated. For the down-regulated transcripts, they mainly belonged in the following categories: translation, stress response, and dehydrogenases. For the translation transcripts, 40% were from ribosomal proteins. There were also two tRNA sythetases (alanine and lysine), two tRNAs (glycine and valine) and a tRNA methyltransferase that were down-regulated. Fifteen dehydrogenase transcripts, which are involved in various aspects of metabolism from lipid metabolism, energy conversion, and amino acid transport, were also down-regulated. Interestingly, the transcript from an aminoglycosidase phosphotransferase (Hlac_0222), which is commonly annotated as a main mechanism of kanamycin resistance ^32^, was also down-regulated. Hlac_0222 was one of the most down-regulated transcripts (-58 fold) in Hla grown in alkaline conditions. It is puzzling why an antibiotic resistance gene would be down-regulated under pH stress; however, it seems clear that the lack of this gene product is important during growth at alkaline pH. While little is know about Hlac_0222, it does have two conserved domains that belong to kinase (cd05154) and phosphotransferases (pfam01636). Therefore, it is possible that Hlac_0222 acts as a general regulator through the transfer of phosphate groups under growth at non-alkaline conditions while this function is down-regulated during growth under alkaline conditions. Transcriptional regulators of the *asnC*, *iclR*, *padR*, *tetR*, and XRE families were also down-regulated.

*Haloferax volcanii* – For *H. volcanii*, microarray analysis showed that transcripts from 326 genes were significantly up-regulated and 1581 were down-regulated at pH 8.0 compared to pH 7.5 ( Table 1). For the up-regulated gene transcripts, the largest percent were members of COG group E (amino acid transport and metabolism) and the lowest percent were members of COG group U (intracellular trafficking, secretion, and vascular transport) and N (cell motility). For the down-regulated gene transcripts, the largest percent were members of COG group E (amino acid transport and metabolism) and the lowest percent were members of COG group A (RNA processing) and B (chromatin structure and dynamics).

As with Hla, the predominant group of up-regulated (58%) and down-regulated (47%) transcripts was from genes without an assigned function (no COG, general function, and unknown function). For the up-regulated transcripts, those with an annotated function mainly grouped as follows: tRNA, metabolism, and stress. For the tRNAs, 31 were regulated from 2- to 1900-fold. For the stress related genes, the small heat shock ( *hsp20* family) and universal stress ( *uspA*) were regulated as was observed in Hla. Additionally, transcripts of the *groEL* chaperone were also up-regulated. For the metabolism, transcripts such as triosephosphate isomerase and beta-glucosidase were significantly up-regulated. For the down-regulated with an assigned function, transcripts from 54 genes are from dehydrogenases, which act in a variety of roles within the cells. Transcripts related to translation were also down-regulated including several tRNA synthetases, tRNA genes, and aminotransferases. Several general transcription factors were also down-regulated including one TATA-binding protein (TBP) and six transcription factor B proteins (TFBs).

*Halobacterium* sp. NRC-1 – For *Halobacterium* sp. NRC-1, microarray analysis showed that transcripts from 471 genes were significantly up-regulated and 72 were down-regulated at pH 8.2 compared to pH 7.2 ( Table 1). For the up-regulated gene transcripts, the largest percent were from COG group J (translation, ribosome structure and biogenesis) and the lowest percent were from COG group U (intracellular trafficking, secretion, and vascular transport). For the down-regulated gene transcripts, the largest percent were from COG group P (inorganic ion transport and metabolism) and H (coenzyme transport and metabolism) and the lowest percent were from COG group C (energy production and conversion) and I (lipid transport and metabolism).

For NRC-1, like Hla and Hvo, the majority of the up-regulated (38%) and down-regulated (61%) transcripts did not have an annotated function. However, for those with an annotation, most of the up-regulated genes were involved in ribosome formation, metabolism and stress. Metabolism genes up-regulated in NRC-1 included aconitase, several cytochromes (b6 and c oxidase), and citrate synthase. Stress genes included superoxide dismutase, *recA*, *rpa*, *uspA*, and *groEL*. For the down-regulated transcripts, they primarily included dehydrogenases. However, the transcript from vng0070h is also of note as it was the most down-regulated transcript (-300 fold). The corresponding gene is annotated to be involved in plasmid stability (COG3668). However, this gene is also a member of COG2026, which contains RelE, which is known to cleave mRNA in the A-site in ribosomes.

In summary, under growth in alkaline conditions, the three haloarchaea studied (Hla, Hvo, and NRC-1) had transcripts from 36 and 15 unique genes (not counting paralogs) in common for the up and down-regulated, respectively ( Dataset 3). This is based on the fact that the genes/transcripts were members of the same COG and/or pfam. Taken together these results show the coordination of 51 unique genes in the same direction (up- or down-regulated) across all three organisms. Specifically, 1.4% of Hla, 1.3% of Hvo, and 2.0% of NRC-1 genes are regulated in the same fashion during growth in alkaline pH.

A majority of these shared transcripts code for products involved in either metabolism (glucose 1-dehydrogenase, malate dehydrogenase, succinate dehydrogenase) or chaperones (small heat shock protein, universal stress protein, Cpn60/TCP-1). Another transcript of note shared by all three organisms belongs to COG 1405 (transcription initiation factor IIB). It has been hypothesized ^24, 33^ that TFBs of archaea act in a similar fashion to the sigma factors of bacteria. Therefore this TFB, most similar to TfbBF in NRC-1 ^24^, regulated in all three organisms, may be the general transcription factor responsible for regulating the response to alkaline pH stress. As seen with *B. subtilis* ^14^ and *E. coli* ^13^ and alkaliphiles ^16^, all three organisms predominantly up-regulated genes involved with metabolism and those that produce acid in response to an alkaline environment.

### Alterations in the transcriptomes under growth in acidic conditions

*Halorubrum lacusprofundi –* For *H. lacusprofundi*, microarray analysis showed that 25 gene transcripts were significantly up-regulated and 43 were down-regulated at pH 6.4, compared to pH 7.4 ( Table 2). For the up-regulated gene transcripts, the largest percent were members of COG group P (inorganic ion and metabolism) and the lowest were members of GOG groups E (amino acid transport and metabolism) and O (post-translational modification, protein turnover and chaperones). For the down-regulated gene transcripts, the largest percent were members of GOG group E (amino acid transport and metabolism) and the lowest were members of GOG group C (energy production and conversion), K (transcription), and T (signal transduction mechanism).

**Table 2.** Transcripts regulated under growth in acidic conditions.

Organism(s)	Number of genes shared Up- regulated	Number of genes shared Down- regulated
Hla	25	44
Hvo	869	987
NRC-1	664	207
Hla and NRC-1	14	7
Hla and Hvo	12	17
NRC-1 and Hvo	213	55
Hla, Hvo, NRC-1	6	7

The majority of the up-regulated (36%) and down-regulated (34%) transcripts did not have an annotated function. However, there were still two genes of interest: hlac_3059 and hlac_3556, which are homologs of *exsB* and *spoVT*. The genes that produce these transcripts are homologs of genes in *B. subtilis* that are up-regulated during periods of spore formation and dormancy ^34, 35^. Hla has not been observed to form spores ^22^, so the regulation of these two genes suggests that the organism is becoming dormant in response to growth in acidic conditions. As with Hvo and NRC-1 (described below), the down-regulated transcripts from Hla were primarily from unknown genes but those with an annotation were primarily from ABC transporters.

*Haloferax volcanii* – For *H. volcanii*, microarray analysis showed that 869 gene transcripts were significantly up-regulated and 987 were down-regulated at pH 6.0 compared to pH 7.5 ( Table 2). For the up-regulated gene transcripts, the largest percent were from COG group E (amino acid transport and metabolism) and the lowest percent were from COG group A (RNA processing and modification) and COG group B (chromatin structure and dynamics). For the down-regulated gene transcripts, the largest percent of transcripts were from COG group E (amino acid transport and metabolism) and the lowest from COG group B (chromatin structure and dynamics).

The largest group of the up-regulated (33%) and down-regulated (46%) transcripts was those without an annotated function. However, for those with an annotation, most of the up-regulated transcripts/genes coded for products involved with stress and motility (e.g. *cct*2, *cct*3, *cspD*3, *cheDFR*, *flaD2J*, and *arcR*1). In Hvo, the *cct* genes form the thermosome, which is a chaperone complex found in archaeal and eukaryotic cells ^36^. It is known for its ability to refold proteins in the cytosol of these organisms. It is also of note that *cct1* was not up-regulated in response to growth in acidic conditions, suggesting that the *cct*2 and *cct3* genes are preferentially used to respond to stress associated with growth in acidic conditions. The up-regulation of the *cspD*3 transcript is also of note for a similar reason as Hvo encodes four paralogous genes. The genes involved with motility: chemotaxis and flagella, were also up-regulated, suggesting that Hvo has a chemotactic response to acidic pH. The up-regulation of the *arcR*1 transcript is interesting as it was up-regulated 7-fold. This is a significant change for a regulator, especially one involved in the control of the arginine metabolism (fermentation) pathway ^37^. Additionally, members of the electron transport chain were also up-regulated, suggesting that a switch in metabolism and energy production is being observed in Hvo during growth in acidic conditions. For the down-regulated transcripts of genes with an annotation, most were transporters, primarily of the ABC-type. ABC-type transporters have been shown to be important in a general stress response in *Listeria monocytogenes* ^38^ as well as in regulation of pH within eukaryal cells and their organelles ^39^. Therefore the down-regulation of these transcripts in Hvo shows that this response is common across all three Domains of Life.

*Halobacterium* sp. NRC-1 – For *Halobacterium* sp. NRC-1, microarray analysis showed that 664 gene transcripts were significantly up-regulated and 207 were down-regulated at pH 5.2 compared to pH 7.2 ( Table 2). For the up-regulated gene transcripts, the largest percent were members of COG group J (translation, ribosomal structure and biogenesis). The lowest percent of transcripts were members of COG group B (chromatin structure and dynamics). For the down-regulated gene transcripts, the largest percent of transcripts were from COG group L (replication, recombination, and repair) and the lowest percent were from COG group M (cell wall, membrane and envelope biogenesis).

The largest group of the up-regulated (24%) and down-regulated (57%) transcripts was that without an annotated function. Many of the up-regulated transcripts in NRC-1, from a gene with an annotated function, coded for products involved with stress, hydrogenases, and motility. Some examples of the up-regulated transcripts were *cctA*, *cctB*, *cspD*2, *dnaK*, *ndhG*1-5, *cheBC1RW2Y*, and *flaA1A2B1B2B3*. The *cctA* and *cctB* genes comprise the thermosome and may be acting in a similar manner as in Hvo to ensure proper protein folding under acidic growth conditions. The *cspD2* gene/transcript is of great interest because it was up-regulated five times more than *cspD*1. Therefore, the difference in *cspD* regulation might suggest that *cspD*2 is specific for acidic stress. The *dnaK* gene is also of interest as its product stabilizes misfolding proteins before being shuttled to the GroEL complex ^40^. The up-regulation of the *ndhG*1-5 genes is also interesting, as an increase in hydrogenases has previously been linked to the acidic pH stress response in *E. coli* and *B. subtilis* ^12– 14^. The up-regulation of genes involved with motility: chemotaxis ( *cheB*, *C*1, *R*, *W*2, *Y*) and flagella ( *flaA*1, *A*2, *B*1, *B*2, *B*3) is also of note as it suggests NRC-1 has a chemotactic response to acidic pH. For the down-regulated transcripts with an annotation, many were transporters such as *trkA*1, *A*2 and ABC transporters (vng795 - a *yufN* homolog). The *trk* genes are responsible for K ^+^ uptake ^2^ and it is possible that the increase in ionic strength associated with the increase in H ^+^ concentration mimics the high salt of the cell enough for it to slow the uptake of K ^+^^41^.

In summary, the responses of Hla, Hvo, and NRC-1 under growth in acidic conditions are quite different to those under alkaline conditions ( Datasets 2, 3, 6–7, 12–15, 18–21). Primarily, there does not seem to be as large of a haloarchaeal specific response. There were six up-regulated and seven down-regulated transcripts in common for all three organisms during growth in acidic conditions ( Dataset 2). For the up-regulated transcripts, they fell into COGs 0614 (ABC-type F3+ hydroxamate transport system) and 0720 (6-pyruvoyl-tetrahydropterin synthase) and pfam 0005 (ABC-transporter), 00528 (transporter system), 01497 (periplasmic binding protein), and 01609 (transposase). For the down-regulated transcripts, they fell into COGs 0589 (universal stress protein A), 1028 (short-chain family oxidoreductase), 1522 (Lrp transcriptional regulator), pfam 00005 (ABC-transporter), 00106 (short-chain dehydrogenase), 00561 (3-oxoadipate enol-lactone hydrolase/4—carboxymuconolactone decarboxylase), and 00582 (universal stress protein family).

A closer look at the response of the three organisms showed that the changes in the transcriptomes of Hvo and NRC-1 were far more similar to each other than either was to Hla ( Dataset 6, 8, 12, 14, 18, 20). Hvo and NRC-1 shared 213 up-regulated transcripts and 55 down-regulated transcripts. For up-regulated transcripts, stress ( *cct* and *cspD*) and motility ( *che* and *fla*) genes were in common. This response closely parallels what has been reported for *E. coli* where heat shock genes were up-regulated in acidic conditions and chemotaxis/flagellar genes down-regulated under alkaline conditions ^42^. For down-regulated transcripts, ABC-transporters were the most common. This shared response accounts for 268 genes or 6.6% of the Hvo and 10.6% of the NRC-1 genes in the respective genomes being regulated in the same fashion.

### Comparison of shifts from one pH extreme to the other

In addition to comparing the extremes of pH to the optimal, we also looked at the changes in transcript abundance from the extremes of pH for each organism. Briefly, this comparison generates a ratio such that a positive value indicates an increase in transcript abundance under a shift from alkaline to acidic conditions and a negative number indicates an increase in transcript abundance under a shift from acidic to alkaline conditions. This comparison is fairly common and has been done routinely for *E. coli* and *B. subtilis* ^13, 15, 42^.

**Acidic to alkaline** – This comparison showed that 20 transcripts ( Table 3) that undergo significant changes in a shift from acidic to alkaline conditions are shared across all three haloarchaea studied ( Dataset 1). Some of the more interesting transcripts that were regulated in common were one general transcription factor (related to *tfbE*), a cell wall/membrane, ABC-transporters, and one replication origin recognition ( *orc*/ *cdc6*). The general transcription factor is of interest as it has been hypothesized that the Tfbs play a primary role is regulating genes, similar to the sigma factors of bacteria ^24^. Therefore, the data strongly suggests that the *tfbE* gene is important in the response in all three organisms when the environmental conditions move from acidic or neutral to alkaline conditions. This is the first evidence from an environmental study in support of this more than ten year-old hypothesis. The *orc*/ *cdc6* gene is of interest for similar reasons. In NRC-1 this gene ( *orc1*) is non-essential; however, like the general transcription factors, the multiple *orc* paralogs found in every haloarchaeon are hypothesized to play a role in alternate start site recognition ^25^. Another interesting note is that a transcript from another *orc gene* ( *orc5*) was regulated under acidic growth possibly providing evidence for alternative replication start sites when growth occurs under pH stress. The ABC-transporters and wall/membrane genes are important, as they possibly constitute a conserved mechanism of the haloarchaea for surviving alkaline conditions, or the shift to alkaline conditions, by a mechanism involving active transport.

**Table 3.** Transcripts regulated under shift from pH extremes.

Organism(s)	Number of genes shared Alkaline to acid	Number of genes shared Acid to alkaline
Hla	417	917
Hvo	2982	231
NRC-1	561	454
Hla and NRC-1	114	98
Hla and Hvo	341	53
NRC-1 and Hvo	508	32
Hla, Hvo, NRC-1	135	20

**Alkaline to acid** – For a shift from alkaline to acidic conditions we observed that 135 of the transcripts ( Table 3 and Dataset 1) are shared across all three organisms. Some of the more interesting genes that were regulated in common were an *orc*/ *cdc6* gene, several ribosomal genes, and a spermidine ABC-transporter. The *orc*/ *cdc6* gene is of interest as this gene in NRC-1 ( *orc5*) is non-essential; however, like the general transcription factors, the multiple *orc* paralogs found in every haloarchaeon are hypothesized to play a role in alternate start site recognition. The *orc5* gene is located on the main chromosome in all three organisms and might play a role in alternative replication start sites under stress conditions. The spermidine ABC-transporter is of interest as spermidine is used as a pH regulator in cells to maintain homeostasis. Its up-regulation in acidic environments suggests that the haloarchaea are responding in a manner similar to many other cells ^43^ from the other domains of life and possibly constitute a conserved mechanism for acidic survival in all three Domains of life.

## Conclusion

The above study is the first to catalog the transcriptomic changes resulting from the growth of haloarchaea in extremes of pH. For this study we used three well-studied haloarchaea: *Halorubrum lacusprofundi* (Hla), *Haloferax volcanii* (Hvo), and *Halobacterium* sp. NRC-1 (NRC-1). We found that under growth in acidic conditions the response of the transcriptomes of Hvo and NRC-1 regulated several gene transcripts in a similar fashion including those for stress and motility. These responses were quite similar to those seen for *E. coli.* However, the response from Hla in general was quite different from Hvo and NRC-1. As a result, it appears that under acidic growth conditions Hvo and NRC-1 respond in a manner similar to other prokaryotes while the response from Hla appears to be species-specific. One interesting variation from the above was the family of ABC-transporters, which were down-regulated in all three haloarchaea. This family is also known to be down-regulated under acidic conditions in other prokaryotes as well as in eukaryotic cells and their organelles. Therefore, it seems that this response is a common response to acidic conditions in all three Domains of Life. For alkaline conditions, the response of the transcriptomes from all three haloarchaea (Hla, Hvo, NRC-1) were very similar to each other and showed that 1.4% of Hla, 1.3% of Hvo, and 2.0% of NRC-1’s transcripts were regulated in the same fashion suggesting a strong haloarchaeal specific response to alkaline conditions. The specific transcripts regulated in Hla, Hvo, and NRC-1 are also regulated in *E. coli*, *B. subtili* *s* and prokaryotic alkaliphiles, strongly suggesting that the haloarchaeal response is also a common prokaryotic response. Finally, our analyses showed that several important genes were regulated under pH stress including *orc*/ *cdc6* and *tfb.* This result was quite interesting as it is an example of one gene with multiple paralogs showing a response under an environmental stress, thereby providing evidence for the hypothesis ^25, 44^ that these multiparalogous genes in the Archaea are used to respond and modulate the response to various environmental stresses.

Data of pH extremes effects on haloarchaea growth and transcriptomic profiles This data file contains 21 datasets. For details on each dataset, please see the Dataset-information text file.

### Data availability

*F1000Research*: Dataset 1. Data of pH extremes effects on haloarchaea growth and transcriptomic profiles, 10.5256/f1000research.4789.d32544 ^45^
