# Supplementary material for: The effects of extremes of pH on the growth and transcriptomic profiles of three haloarchaea
Source: F1000Res. 2014 Aug 29;3:168. Originally published 2014 Jul 22. [Version 2] doi: 10.12688/f1000research.4789.2 (PMC4176423; doi:10.12688/f1000research.4789.2)
Supplement: Peer review history

# Peer review: Referee response for version 2

**DOI:** 10.5256/f1000research.5486.r5993

**Published:** 2014-09-18

**Authors:** Jesse Dillon

**Affiliations:** 1 Department of Biological Sciences, California State University, Long Beach, USA

I now approve the revised manuscript.

I have read this submission. I believe that I have an appropriate level of expertise to confirm that it is of an acceptable scientific standard.

# Peer review: Referee response for version 1

**DOI:** 10.5256/f1000research.5113.r5553

**Published:** 2014-08-14

**Authors:** Melanie Mormile

**Affiliations:** 1 Department of Biological Sciences, Missouri University of Science and Technology, Rolla, MO, USA

This is the first study to determine the transcription response of haloarchaea when grown under extreme pH conditions.  It is surprising that the pH tolerances of the select organisms have not been determined before and it was good to see that the authors did determine the pH values each of the organisms could tolerate.  This data enabled the authors to determine the up-regulation and down-regulation of individual genes under the most acidic and alkaline conditions the organisms could tolerate in comparison to optimal pH conditions.  The results support the authors' conclusions. The only thing that I would like to see included is a more complete discussion of how their results support their statement "We also chose three organisms, as opposed to one, to ascertain if the response to pH was specific to the organism, the Halobacteriaceae, the Prokaryotes, or the three Domains of Life." in the first paragraph of the Results and Discussion.  The authors touch on this a few times in the article but do not really bring it to a full conclusion.

I have read this submission. I believe that I have an appropriate level of expertise to confirm that it is of an acceptable scientific standard.

# Response

**Published:** 2014-08-15

**Authors:** James Coker

**Affiliations:** The Graduate School, University of Maryland, University College, USA

Thank you very much for you comments.  We have added a bit more discussion at the end of the introduction, in the results and discussion section, and in the conclusion section about why the three organisms were selected and the nature of their transcriptomic responses.

# Peer review: Referee response for version 1

**DOI:** 10.5256/f1000research.5113.r5557

**Published:** 2014-08-12

**Authors:** Mohammad Ali Amoozegar

**Affiliations:** 1 University of Tehran, Tehran, Iran

The paper “The effects of extremes of pH on the growth and transcriptomic profiles of three haloarchaea” by Moran-Reyna and Coker describes effects of pH on growth of three genera in extremely halophilic archaea . The authors of the paper are expert in halophilic world studies. The manuscript is well-written and is quite straightforward. The language is very good. The paper was generally well structured and the results well implemented and presented.

Please consider the following comments:

1. Please add name of strains for three haloarchaea species.
2. please add a conclusion for the manuscript.      

I have read this submission. I believe that I have an appropriate level of expertise to confirm that it is of an acceptable scientific standard.

# Response

**Published:** 2014-08-15

**Authors:** James Coker

**Affiliations:** The Graduate School, University of Maryland, University College, USA

Thank you very much for you comments.  We have added the names of the strains used in our studies to the materials and methods section.  We have also added a conclusion section to the manuscript.

# Peer review: Referee response for version 1

**DOI:** 10.5256/f1000research.5113.r5552

**Published:** 2014-08-05

**Authors:** Jesse Dillon

**Affiliations:** 1 Department of Biological Sciences, California State University, Long Beach, USA

This manuscript describes a study of responses of three well-characterized halorachaea to pH changes. Specifically, growth changes were measured and transcriptional changes were measured via cDNA microarray analysis for cells placed under different pH treatments. The authors observed some interesting differences in responses, especially in the *Halorubrum* strain under acidic conditions suggesting that pH responses are not universal among Halobacteriales. The finding that TFB, a putative transcription factor, is universally found under alkaline conditions may be of importance in understanding general responses to pH in haloarchaea. Overall, the manuscript is well written and the science seems sound; however, additional information in various sections of the manuscript is necessary to fully evaluate the study.

**Abstract:**

- The statement " *However, of all the naturally occurring stresses faced by the haloarchaea, only one, pH, has not been previously reported on*." is incorrect as a number of studies have reported on pH optima for described halorachaea. For example, Bowers and Wiegel published a minireview on pH and temperature responses of haloarchaea ( Bowers and Wiegel, *Extremophiles,* 2011). I would recommend the authors qualify this statement to indicate they are the first to measure gene transcript changes in responses to pH and also include this reference and others from the Wiegel group in their article.
- Although the shorthand abbreviations for halophiles are well known within the halophile research community, I would define the three species using full scientific names on first usage in the abstract since F1000 has a very general audience.
- The statement beginning "Our observations..." could be improved by specifying the similarities or differences. As written the sentence is more indicative than substantive.

 

**Introduction:**

- The extensive summary of results presented in the introduction beginning "Broadly..." is inappropriate in the introduction and should be deleted.

 

**Methods:**

- More detail is needed on how the various culture media were adjusted for pH. What buffers were used? How did the authors ensure that pH did not change throughout the growth experiments?
- The way the description of pH manipulations is written made it seem like the optimal pH 7.2-7.5 control cultures were not included, although that is not the case based on their results. The authors should include these values in the list of treatments.
- Reference is made to control samples in the cDNA preparation section. Please elaborate on exactly what these controls were and how they affected the study's findings.
- It would be helpful if the Perl scripts described in the Microarray data processing section were made available in the supplementary information section.
- I may be missing something here. Reference is made to " *genes showing greater than 2-fold change*" in transcriptional level in " *at least" 2 replicates”*. However, the methods describe duplicate technical microarray replicates, so how could it be more than 2?  Please clarify.

 

**Results/Discussion:**

- Since optical densities vary among strains -- it would be useful for comparative purposes if the authors could calculate and report their growth data in terms of cells/ml as opposed to OD.
- What do the error bars represent in the growth curve figures? Overall, the degree of replication in the growth experiments is unclear, please clarify the statement: " *Each growth curve was measured twice and replicated once*."
- Statistical tests (e.g. ANOVA) for differences among growth curves should be performed to better support statements about differences in growth rates.
- The section Microarray analysis and responses in R/D might be better placed in methods since it does a good job of clarifying the methodology and does not really report findings.
- Some interpretation of their results in the broader ecological context of pH environments encountered in nature would be warranted. For example, in the introductions, the authors note that halophiles have been isolated from sites with extremes in pH; however, their current study focuses on well characterized, "lab rat" haloarchaea isolated from circumneutral habitats. These strains have the advantages of characterized genomes, etc. However, in future studies it would be interesting to see if the same responses are found for organisms such as alkaliphiles isolated from more extreme pH habitats. Acknowledgment of these limitations/opportunities in the discussion would be appropriate.

 

**Presentations of Figures:**

- Presentation of figures would be improved if the authors matched error bar colors with the line. Also, the small size of line markers and lack of contrast among some of the colors (e.g. dark shades of blue and black) makes it hard to differentiate some of the growth curves.

I have read this submission. I believe that I have an appropriate level of expertise to confirm that it is of an acceptable scientific standard, however I have significant reservations, as outlined above.

# Response

**Published:** 2014-08-15

**Authors:** James Coker

**Affiliations:** The Graduate School, University of Maryland, University College, USA

Thank you very much for your thoughtful recommendations.  We have incorporated all but one of them into the manuscript.  The only suggested change not made was to report the growth curves as cells/ml.  We have instead kept the OD readings as they are since this is a very standard way of reporting this type of data.  We understand the issue of cell size possibly affecting the OD readings; however for the haloarchaea this is correlated with the production of gas vesicles, which are typically produced during cell densities (i.e. anaerobic conditions (OD600 >= 2.0)) well below the ones we report here.
